# The Impact of the Fungal Priority Pathogens List on Medical Mycology: A Northern European Perspective

**DOI:** 10.1093/ofid/ofae372

**Published:** 2024-07-01

**Authors:** Maiken Cavling Arendrup, Darius Armstrong-James, Andrew M Borman, David W Denning, Matthew C Fisher, Rebecca Gorton, Johan Maertens, Ignacio Martin-Loeches, Varun Mehra, Toine Mercier, Jessica Price, Riina Rautemaa-Richardson, Rachel Wake, Natalie Andrews, P Lewis White

**Affiliations:** Unit of Mycology, Statens Serum Institut, Copenhagen, Denmark; Department of Clinical Microbiology, Rigshospitalet, Copenhagen, Denmark; Department of Clinical Medicine, University of Copenhagen, Copenhagen, Denmark; Department of Infectious Disease, Imperial College London, London, UK; Mycology Reference Laboratory, UK Health Security Agency, Bristol, UK; Medical Research Council Centre for Medical Mycology, University of Exeter, Exeter, UK; Manchester Fungal Infection Group, The University of Manchester, Manchester, UK; Global Action For Fungal Infections, Geneva, Switzerland; MRC Centre for Global Infectious Disease Analysis, Imperial College London, London, UK; Department of Infection Sciences, Health Services Laboratories, London, UK; Department of Hematology, University Hospital Gasthuisberg, Leuven, Belgium; Department of Intensive Care Medicine, St. James's Hospital, Dublin, Ireland; Hospital Clinic, IDIBAPS, Universidad de Barcelona, Spain; CIBERes, Barcelona, Spain; Department of Haematological Medicine, Kings College Hospital NHS Foundation Trust, London, UK; Department of Oncology-Hematology, AZ Sint-Maarten, Mechelen, Belgium; Department of Microbiology, Immunology, and Transplantation, KU Leuven, Leuven, Belgium; Department of Hematology, University Hospitals Leuven, Leuven, Belgium; Public Health Wales Mycology Reference Laboratory, UHW, Cardiff, UK; Department of Infectious Diseases, Manchester University NHS Foundation Trust, Wythenshawe Hospital, Manchester, UK; Division of Infection, Immunity and Respiratory Medicine, School of Biological Sciences, NIHR Manchester Biomedical Research Centre (BRC) at the Manchester Academic Health Science Centre, The University of Manchester and Manchester University NHS Foundation Trust, Wythenshawe Hospital, Manchester, UK; Mycology Reference Centre Manchester (MRCM), ECMM Excellence Centre of Medical Mycology, Manchester University NHS Foundation Trust, Wythenshawe Hospital, Manchester, UK; Institute for Infection and Immunity, St George's University of London, London, UK; Napp Pharmaceuticals Limited, a member of the Mundipharma network of independent associated companies, Cambridge, UK; Public Health Wales Mycology Reference Laboratory, UHW, Cardiff, UK

**Keywords:** fungal diagnostics, fungal surveillance, mycology, WHO fungal priority pathogen list

## Abstract

Fungal diseases represent a considerable global health concern, affecting >1 billion people annually. In response to this growing challenge, the World Health Organization introduced the pivotal fungal priority pathogens list (FPPL) in late 2022. The FPPL highlights the challenges in estimating the global burden of fungal diseases and antifungal resistance (AFR), as well as limited surveillance capabilities and lack of routine AFR testing. Furthermore, training programs should incorporate sufficient information on fungal diseases, necessitating global advocacy to educate health care professionals and scientists. Established international guidelines and the FPPL are vital in strengthening local guidance on tackling fungal diseases. Future iterations of the FPPL have the potential to refine the list further, addressing its limitations and advancing our collective ability to combat fungal diseases effectively. Napp Pharmaceuticals Limited (Mundipharma UK) organized a workshop with key experts from Northern Europe to discuss the impact of the FPPL on regional clinical practice.

## Teaser

Fungal diseases represent a considerable global health concern. In 2022, the World Health Organization introduced the pivotal fungal priority pathogens list. Here, the impact of it is discussed, covering topics such as diagnostics, surveillance, education, guidelines and one health.

Fungal diseases represent a widely overlooked global public health concern. They are known to affect >1 billion people globally and are responsible for >2.5 million annual deaths [1, 2]. Factors contributing to the increase in global incidence of fungal diseases include an ever-growing population that is susceptible to fungal diseases, environmental change, and increased international travel/trade, with increasing antifungal resistance (AFR) complicating disease management and leading to increased morbidity and mortality [3–5]. Fungal pathogens belonging to the *Aspergillus*, *Candida*, *Cryptococcus*, and *Pneumocystis* genera account for >90% of reported fungal-related deaths, but other fungal diseases have also been implicated in specific clinical scenarios associated with high disease burden (eg, mucormycosis in coronavirus disease 2019 [COVID-19]) [6–8]. Large-scale fungal outbreaks with a range of pathogens are also increasingly being documented as causing a rise in antifungal resistance (*Pneumocystis pneumonia, C. auris* infection, and *C. parapsilosis*), including less common fungal pathogens (eg, *Exserohilum rostratum*) [9–11]. They all represent a significant infection prevention and control dilemma and are associated with significant morbidity and mortality [9, 12]. Beyond species capable of causing invasive fungal diseases (IFDs; fungal pathogens infecting the blood and/or tissues/organs), the emergence of contagious and virulent dermatophyte species such as *Trichophyton rubrum, Trichophyton mentagrophytes,* and *Trichophyton indotinieae* is particularly concerning given these species’ antifungal resistance profiles [13, 14].

Historically, patient populations at high risk of developing IFDs have included patients who are immunosuppressed (eg, primary immunodeficiencies), patients with neutropenia during acute leukemia or post­–allogeneic stem cell transplantation, solid organ transplant recipients, patients in the intensive care unit, patients undergoing abdominal surgery, and patients with late-stage HIV infection [4, 15]. However, the growing use of corticosteroids and immunomodulatory therapy is rendering more people susceptible to chronic fungal diseases and IFDs [4, 15]. Infection with fungal pathogens during clinical procedures, after natural disasters, and during conflict is leading to an increase in documented IFDs in immunocompetent patients [16, 17]. It is currently unclear how environmental change will impact the natural geographical distribution of fungi, but it may be particularly relevant for endemic fungi (*Coccidioides* spp., *Histoplasma* spp., *Blastomyces* spp., *Talaromyces marneffei, Paracoccidioides* spp., and *Sporothrix* spp.) that present a significant disease burden within their current endemic regions [18, 19]. Environmental change has been postulated to have contributed to the emergence of *C. auris* in particular [18–20].

Currently, there are 4 main clinically licensed classes of antifungal agent: azoles, echinocandins, pyrimidines, and polyenes [12]. The modest number of available antifungal drug classes is compounded by resistance to these treatments, with the presence of potentially multidrug-resistant species (eg, *C. auris, C. glabrata, C. krusei,* and *C. parapsilosis*) and both environmentally and clinically driven AFR (eg, *A. fumigatus* and *A. flavus*) having been found to exhibit high resistance to azoles and amphotericin B [12, 21–23].

In late 2022, the World Health Organization (WHO) published the first fungal priority pathogens list (FPPL), containing 19 fungal species [12]. Fungi associated with a spectrum of clinical manifestations that threaten public health are the focus of this list, with the priority pathogens identified by assessing clinical need against research requirements [12, 24]. The aim of the list was to “focus and drive further research and policy interventions to strengthen the global response to fungal diseases and AFR” [12]. The initial fungal pathogens of concern were chosen based on international recommendations; subsequent systematic reviews determined the prevalence of disease and AFR for each pathogen [12, 24]. Ten semiquantitative criteria were agreed upon, and the pathogens were ranked using a discrete choice experiment involving >300 practitioners and diagnostic laboratories; the pathogens were then categorized into 3 priority groups: critical, high, and medium risk [12, 24]. The FPPL represents the first global initiative to prioritize the management of fungal pathogens, recognizing the global public health impact, and highlighting unmet needs [12]. There is a clear need for global evidence-based public health interventions that enhance research and development (R&D) and improve surveillance; such interventions are highlighted and discussed in this manuscript [12].

## METHODOLOGY

A collaborative workshop with key experts from Northern Europe was held in July 2023 to discuss the impact of the FPPL on regional clinical practice. The initiative included representatives of various specialties, including infectious diseases, intensive care, respiratory medicine, hematology, microbiology, mycology, and fungal epidemiology, from Denmark, the United Kingdom, Belgium, and Ireland. Invited experts reflected the regional scope, but given the broad and extensive knowledge available, many of the outputs are still applicable outside this region. During the workshop, presentations on the key topics described below were provided to stimulate debate and individual opinion, which was subsequently documented. This manuscript was developed by integrating workshop discussions with supporting literature findings. Additional comments and observations during the workshop were captured to supplement specific sections and/or statements.

### Impact of the FPPL in Low- and Middle-Income Countries

Without quality diagnostics, the prevalence of all fungal diseases is underestimated [12]. In 2018, the WHO established the Model List of In Vitro Diagnostics, which supports low- and middle-income countries (LMICs) in improving access to diagnostics. The list includes microscopy, fungal culture, blood culture, histopathology, cryptococcal, *Histoplasma,* and *Aspergillus* antigen, *Aspergillus* antibody, and *Pneumocystis* real-time polymerase chain reaction (PCR) [25–27]. Unfortunately, many countries have no or limited access to some or all of these diagnostics [12, 28–33]. Antifungal susceptibility testing (AST) and antifungal therapeutic monitoring are usually available in high-income countries but are rarely done in most LMICs [12, 28–33]. Appropriately, the WHO FPPL focuses on the need to strengthen diagnostic capacity, not only for patient care but also as a necessary requisite for surveillance [12]. The true burden of fungal diseases and AFR will be impossible to ascertain without improved diagnostic coverage [12].

The lack of mycology laboratories and expertise in many LMICs has also led to low awareness of fungal diseases among health care professionals (HCPs) [34–38]. The overall incidence of health care–associated infections, such as candidemia, is considerably high in many middle-income countries, likely reflecting incomplete infection control efforts, excess antifungal use, and few stewardship programs [38]. To alleviate fungal disease burden in these countries, it is important to focus on identification and appropriate testing in high-risk groups, for example, cryptococcal and *Histoplasma* antigen testing in hospitalized patients with HIV, *Aspergillus* immunoglobulin G (IgG) antibody testing in patients with chronic chest symptoms, and blood culture in hospitalized febrile patients with possible sepsis [38–41].

The WHO has highlighted a stepwise approach to assist countries in improving mycology diagnostic capacity, addressing the need to manage fungal diseases and perform surveillance. This has underpinned global roadmaps, focusing on tests (PCR, antigen/antibody testing) that require less training due to their speed and simplicity, to quickly improve diagnostic capacity [12, 25, 42]. Culture-based methodologies are important because they allow for pathogen identification and susceptibility testing, but they can lack sensitivity [25, 43].

The WHO recommends implementing the FPPL incrementally, starting with the highest-priority pathogens (and their associated diagnostic tests), generating data on fungal pathogens, and subsequently adjusting the FPPL to align with regional, national, and local needs; this will be critical in LMICs, especially in the presence of endemic fungi and high rates of HIV infection [12].

### Impact of the FPPL on Diagnostics

Early and accurate diagnosis is crucial in the effective treatment of fungal diseases [44]. Diagnosis can be challenging due to the limitations of current methodologies, including low sensitivity and specificity in certain settings, which can result in missed or inaccurate diagnoses [44, 45]. Lack of timely access to high-quality diagnostics and optimal treatment regimens and the potential evolution of AFR in some settings present further challenges in managing fungal diseases [12]. Conventional methodologies, such as culture, microscopy, and histopathology, form the basis of fungal disease diagnosis and play a vital role in the diagnosis of nearly all fungal diseases [44–47].

Guidelines developed by the European Confederation of Medical Mycology (ECMM), the International Society for Human and Animal Mycology, the American Society for Microbiology, and the Mycoses Study Group Education and Research Consortium recommend microscopy and culture in the diagnosis of IFDs [7, 32, 48, 49]. However, conventional methodologies generally require a high level of experience in identifying fungi [44, 46, 50]. While microscopy can yield rapid preliminary results, culture normally takes at least 24–48 hours to provide a positive result and can take up to 30 days [44, 46, 50–53]. As a result, there is increasing research on alternative ways to identify IFDs (Table 1). A survey conducted by the ECMM in 2021, which was designed to assess IFD diagnostic capacity in Europe, showed an overall acceptable level [32]. Of the 388 participating institutions from 45 countries, 99% had access to culture-based methodologies, 97% to microscopy, 94% to antigen-detection assays, 85% to molecular tests, and 84% to antibody tests [32]. According to the gross domestic product (GDP) per capita of each European country, access to methodologies varied considerably, with the exception of microscopy [32]. Antigen detection tests were also more readily available in countries with a GDP per capita of ≥$30 000 (95%–96%) compared with countries with a GDP per capita of <$30 000 (83%) [32].

**Table 1.** Current Diagnostic Methodologies Used in Mycology Laboratories in Diagnosis of Fungal Pathogens Highlighted in the FPPL^a^ [7, 12, 19, 44–46, 54–76]

	Blood Culture	Fungal Culture	β-D-Glucan Testing	Galactomannan	Commercial PCR^b^	Antigen^c^	Antibody^c^
Critical group							
*Cryptococcus neoformans*^d,e^	+	+	+/−	−	+	+	+
*Candida auris*^f^	+	+	+	−	+	+/−	+/−
*Aspergillus fumigatus*^e^	−	+	+	+	+	+	+
*Candida albicans*^f^	+	+	+	−	+	+	+
High group							
*Candida glabrata* (*Nakaseomyces glabratus*)^f^	+	+	+	−	+	+/−	+/−
*Histoplasma* spp.^e^	+	+	+	+/−	In-house	+	+
Eumycetoma causative agents	−	+	−	−	In-house	−	−
Mucorales^g^	−^h^	+	−	−	+	−	−
*Fusarium* spp.	+	+	+	+	In-house	−	−
*Candida tropicalis*^f^	+	+	+	−	+	+/−	+/−
*Candida parapsilosis*^f^	+	+	+	−	+	+	+
Medium group							
*Scedosporium* spp.	−	+	+	−	In-house	−	−
*Lomentospora prolificans*	+/−	+	+	−	In-house	−	−
*Coccidioides* spp.	+/−	+	+	+/−	In-house	+	+
*Candida krusei* (*Pichia kudriavzevii*)^f^	+	+	+	−	+	+/−	+/−
*Cryptococcus gattii*^d,e^	+	+	+/−	+/−	+	+	+
*Talaromyces marneffei*	+	+	+	+/−	In-house	+^i^	−
*Pneumocystis jirovecii*	−	+/−	+	−	+	−	−
*Paracoccidioides* spp.	−	+	+	+/−	In-house	−	In-house

+, detected in infections; −, not detected in infections; +/−, can be detected in some infections.

Abbreviations: FPPL, fungal priority pathogens list; PCR, polymerase chain reaction.

^a^The sensitivity and specificity of the tests listed in the table vary and have not been validated in all specimens.

^b^Excluding pan-fungal PCR assays.

^c^Antigen and antibody tests will likely have been optimized against the most prevalent fungal species (eg, *C. albicans* or *Aspergillus fumigatus*) and assay performance when detecting other species may be compromised.

^d^Latex agglutination is also available as a diagnostic method.

^e^Lateral flow assay/device is also available as a diagnostic method.

^f^CHROMagar *Candida* Plus is also available as a diagnostic method.

^g^Commercial PCR testing is not available for all Mucorales spp.

^h^Except for *Mucor circinelloides*.

^i^The mAb-Mp1p enzyme immunoassay was approved for use in China in October 2019. It is also being evaluated in 1 prospective multicenter study (NCT04033120).

While most European countries are well equipped to manage fungal diseases, some institutions lack access to specific diagnostics and antifungal medications; this needs to be addressed if fungal disease management is to improve [32]. Molecular tests may be more rapid and may be more sensitive than classical microbiologic tests in some situations, but they have limitations, such as accessibility, variable standardization, and commercial options [50, 77–80].

There have been some efforts to increase fungal diagnostic capacity and decrease turnaround times, including the development of lateral flow assays with the potential for point-of-care testing [19, 45, 81]. However, there are few molecular and serological tests that are widely commercially available [19, 45]. The FPPL recommends improving fungal disease surveillance and access to affordable diagnostics, which will hopefully improve diagnostic access/capacity across Europe, but particularly in countries with a low GDP per capita [12, 24].

### Impact of the FPPL on Surveillance

AFR is a growing global public health concern leading to increased morbidity and mortality [12]. Acquired azole resistance to the following pathogens is increasing: *Aspergillus fumigatus* (globally, where surveillance has been performed), *C. parapsilosis* (in several European countries, including Croatia, Greece, Romania, Russia, Spain, and Turkey), and *C. tropicalis* (primarily in Asia) [82–90]. This is alarming as these pathogens can cause IFD. Acquired terbinafine resistance is also rapidly emerging in *Trichophyton* spp., and although these fungi do not cause life-threatening infections, recalcitrant tinea corporis can impair quality of life [91]. Rates >30% have been described in India due to *T. indotineae* resistance; this species, along with terbinafine-resistant *T. rubrum*, is now increasingly being reported in Europe, Africa, and the United States [92–94]. However, owing to the lack of effective fungal disease surveillance and limited laboratory capacity in many countries, the global impact of AFR is unknown [12]. Nationwide surveillance data for *Candida* spp. are accessible (but not always systematic) in countries such as Australia, Denmark, Finland, Iceland, Norway, Scotland, Sweden, and the United Kingdom; however, surveillance data for other fungal species are not as common, as most published data are limited to azole-resistant *Aspergillus fumigatus* [21, 54, 95–98]. Nationwide data for azole-resistant *Aspergillus fumigatus* are available in countries such as Belgium, Denmark, Greece, the Netherlands, and Spain [99–103].

The English surveillance program for antimicrobial utilization and resistance has collaborated with external stakeholders to ensure effective antimicrobial administration and resistance surveillance, as well as continued delivery of antimicrobial stewardship programs [104]. Routine laboratory surveillance reports are submitted to the UK Health Security Agency [104]. The WHO is incorporating fungal pathogens into their existing surveillance system, known as the Global Antimicrobial Resistance and Use Surveillance System, which was implemented in 2019 and is the first global collaborative effort to standardize antimicrobial resistance surveillance [12, 105].

For the FPPL to fulfil its aims, there is a clear need to strengthen evidence on R&D, public health interventions, and surveillance. Broadening access to mycology laboratories is crucial in enhancing surveillance strategies and ensuring optimal patient care and safety [12]. High-quality diagnostics for IFDs are essential to the WHO AFR strategy [12]. To achieve this, attention should be given to 5 areas: using reference microbiology laboratories for surveillance and training; integrating fungal diagnostics into routine or specialized health care; limiting inappropriate use of antifungal agents through stewardship programs; promoting the development of national and international networks for knowledge transfer and research; and facilitating large-scale data collection on susceptibility testing to aid in the development of clinical breakpoints [12]. In addition, integrated systems to enable the surveillance of fungal pathogens across the environment and animal and human health will be essential to better understand the drivers for AFR [21]. These measures would collectively contribute to improvements in surveillance and patient outcomes [12].

AST is a technique used in clinical microbiology laboratories that aims to guide patient care and track rates of AFR [106]. Although there is limited information on the global availability of AST methods, which may complicate international resistance surveillance initiatives, the Clinical and Laboratory Standards Institute in the United States and the European Committee on Antimicrobial Susceptibility Testing (EUCAST) in Europe have established standards for susceptibility testing and determining clinical breakpoints [12, 21, 107]. The clinical breakpoints determined by EUCAST classify organisms as S (susceptible), I (susceptible, increased exposure), or R (resistant). Breakpoints have been established for several species, including *Candida* spp. (amphotericin B, echinocandins, and azoles), but can be limited for some species such as *Aspergillus* spp. (amphotericin B and azoles) and *Cryptococcus neoformans* (amphotericin B) [107, 108]. This has not been achieved in less common species owing to a lack of sufficient data, particularly regarding the relationship between minimal inhibitory concentration and clinical outcome; however, pragmatic guidance has been provided by EUCAST for >30 rare yeasts [22, 107]. Breakpoint application relies on precise species-level identification and has been improved for yeasts, using MALDI coupled to time-of-flight (MALDI-TOF) mass spectrometry methodologies; however, for molds, it is still reliant on local databases [21]. Although MALDI-TOF is too expensive for many centers, direct detection of AFR using this method is an exciting advancement [21]. We therefore recommend optimizing and revising AST methods, which will be crucial for many centers and countries, especially those that cannot incorporate, access, or afford the current tests. The FPPL could be critical in the development of easily accessible testing or reinforcing the need for financial support to provide the recommended methods [106].

Furthermore, genomics can be used as a tool to understand the molecular epidemiology of resistance or as a potential diagnostic tool for fungal isolates [109, 110]. For example, whole-genome sequencing of *C*. *auris* infections from the largest UK outbreak to date, which occurred between April 2015 and November 2016, uncovered multiple antifungal-resistant *C. auris* genotypes [109].

The development of the FPPL has highlighted the importance of standardizing surveillance system reports; this will enhance the connection between surveillance and antifungal susceptibility [12, 21]. It is important to improve coverage of surveillance data for fungal diseases to accurately determine disease burden, assess R&D priorities, and make recommendations for stewardship programs [21, 111]. Overall, improved surveillance relies on an adequate level of diagnostics, knowledge, and education regarding clinical presentation and risk factors for infection with these pathogens [12].

### Impact of the FPPL on Education of Clinicians and Infection Specialists

One of the main challenges in implementing the FPPL is the poor coverage of fungal diseases in medical curricula and the limited number of clinicians and infection specialists with expertise in fungal diseases [12, 112, 113]. This knowledge gap is also partially due to limited surveillance and the sporadic provision of fungal diagnostics, which contribute to clinicians' limited practical experience in interpreting fungal diagnostic test results and targeted management of fungal diseases [12, 105]. Publication of the FPPL is an important first step in raising awareness of fungal pathogens and the need for data and evidence generation [12, 24]. We recommend persuading those in charge of undergraduate and postgraduate medical curricula to expand the learning objectives beyond empiric management of the most common mycoses and guide the development of the curricula for allied health professions, such as biomedical and clinical scientists.

It is important to acknowledge that the diagnosis and management of fungal diseases have specific features that differ from other infections (eg, bacterial) [114]. Learning a completely new concept is not easy if the foundations are weak; therefore, the educational approaches targeted at practicing clinicians need to take this into consideration. The education of practicing clinicians also needs to be tailored to their environment and the services available to them, focusing on how these diagnostics can be best utilized. At the same time, we recommend joint training for clinicians and laboratory technicians to ensure that all stakeholders are aware of the tests available to them and that the laboratory team gains further insight into clinical requirements/demands.

Trainee infection specialists should be advised on how to write business cases for the introduction of new diagnostic tests recommended in global guidelines and how to tackle bottlenecks, such as poor turnaround times, for instance by liaising with diagnostic laboratories [46].

Overall, acknowledgement by the WHO that various fungal diseases have been overlooked and the introduction of the FPPL have considerably raised awareness in this area [115]. These steps are key to improving education and increasing knowledge of health care providers and scientists on the importance of combatting IFDs [115].

### Impact of the FPPL on the Education of Research and Health Care Scientists

Within general diagnostic microbiology laboratories, bacteriology and virology remain the main foci, with mycology (outside of specialist reference centers) limited to classical microbiology testing; this is reflected in the range and depth of education provided to health care scientists [116, 117]. This is contrary to the information provided by the FPPL, which defines fungal diseases as more than just a skin or nail infection [12]. Increasing the academic availability of this subject through training programs would encourage scientists to specialize in mycology, which would enhance research development in this subject [118]. Within specialist mycology laboratories, both research and diagnostic testing are performed, allowing staff to gain experience in tests, but this is not currently reflected in general educational programs [119].

When working within a specialist mycology setting, progression can be limited by the lack of knowledge on this subject in standard microbiology educational programs. Alternative routes of accreditation (eg, equivalence routes), which assess the current level of education and experience of the candidate while they maintain their current work commitments, can be more onerous than the generic route. Within academia, research roles are generally governed by the availability of grant funding and tenured posts within mycology and are less available than in other areas of microbiology [115, 120]. Subsequently, there is no ideal option for training, retaining, and progressing scientific staff with a desire for a career in mycology, and experienced staff regularly apply for non-mycological jobs to advance their careers in a more timely manner.

The FPPL has the potential to enhance awareness of the diagnostic and research possibilities within mycology, but more must be done to stratify the educational processes to retain and attract staff with an interest in mycology [12, 21, 24]. The FPPL could be used to encourage collaboration between health educational institutions and experts in the mycological field to develop a program that enables scientists to gain in-depth education in mycology [12, 24]. Indeed, a multidisciplinary approach to researching fungal pathogens can provide a starting point to identify current knowledge gaps and establish collaborative networks among researchers [19].

### Impact of the FPPL on Mycology Guidelines

Due to the complexity of patient groups who are at risk of developing fungal diseases and the relatively low incidence of proven disease, clinical trials have focused on specific patient groups in hemato-oncology [12, 121]. Recent clinical trials in more common superficial *Candida* infections have highlighted the diagnostic challenge of distinguishing colonization from infection [122]. A lack of research funding to tackle these challenges or update epidemiological data has not helped [1, 115, 120]. Therefore, many aspects of fungal guidelines are based on expert opinion, potentially extrapolating data from one specific cohort to another [123]. Subsequently, the strength of recommendation is high, but the quality of evidence is lacking [12, 121].

The guidelines for fungal diseases have been developed by different groups: the European Society of Clinical Microbiology and Infectious Diseases (ESCMID), the Fungal Infection Study Group, the Infectious Diseases Society of America, the ECMM, the European Conference on Infections in Leukaemia, and the International Society for Human and Animal Mycology [7, 108, 124]. These guidelines provide direction on diagnostics and procedures for various pathogens, including those in the FPPL critical-priority group [108]. Guidelines such as those developed by ESCMID can be used to support national societies in developing and strengthening local guidance for tackling fungal diseases [125]. However, national microbiology protocols often focus on the recovery of nonfungal pathogens, and incorporating international recommendations into generic protocols can be hindered by logistical and financial issues, although the necessity to make such changes is supported by the FPPL [12, 24, 46]. HCPs must use their expertise and undertake treatment decision-making on a case-by-case basis; they must also stay up to date with data and evidence, which may not be highlighted in the guidelines at the time of their development. The FPPL emphasizes the significance of fungal diseases to HCPs and rates each fungal disease according to significance and potential need for guidelines, if lacking [126].

Assessing clinical adherence to current guidelines can be challenging [126]. The ECMM introduced the ECMM Quality (EQUAL) of Clinical Management score to provide straightforward yet comprehensive guidance for physicians and quantify adherence to guidelines as a proxy for assessing the quality of diagnostics and therapeutic care [127]. These scores are currently used for candidemia, invasive aspergillosis, cryptococcosis, mucormycosis, fusariosis, and *Scedosporium*-, *Lomentospora*-, and *Trichosporon*-associated infections; adherence to the guidelines has been associated with improved patient care [127, 128].

Strategies within the FPPL recommendations include improvement of health care systems to promote evidence-based therapy, diagnosis, AFR detection, and AFS programs, which can be underpinned by available guidelines [12, 126]. Clinical decision-making regarding antifungal treatment can be aided by efficient stewardship programs, which will enhance patient outcomes and reduce health care costs [126]. Those responsible for AFS should be knowledgeable and skilled in treating fungal diseases, and physicians who are involved in stewardship should inform other health care providers on the proper use of antifungal agents to optimize patient care [126].

### Impact of the FPPL on One Health

Humans are exposed to opportunistic pathogenic fungi on a daily basis, as these fungi are frequently found in our environments. Many are known to produce abundant airborne spores, and some of the FPPL pathogens fall into the “opportunistic” category owing to their sporulating lifestyles [12, 21]. The health impact caused by exposure to opportunistic pathogenic fungi is not equally distributed across populations [19]. First, environmental niche is critical to determining exposure; for example, several of the FPPL fungal species are typically not found in Europe as they are endemic in other regions [12, 19, 129, 130]. Second, risk factors are unequally distributed, with demographic, socioeconomic, occupational, and spatial factors all determining the health impact of exposure to FPPL pathogens [19]. Third, large-scale anthropogenic processes, such as farming practices, land use change, and climate change, all play a role in the ecology of FPPL pathogens, with consequential effects on human exposure [9]. Taking these arguments into account, it is clear that understanding the health impact of FPPL fungal pathogens at a granular level requires an integrated approach that includes wider environmental factors; such unified system-level understanding is often known as “One Health” [21, 131].

One Health programs that tackle disparities in social issues, such as occupation, housing, climate change, combat-related injuries, and health care access, are likely to play a role in limiting fungal diseases [18, 19, 132]. However, the evidence base illustrating health improvements against fungal diseases is nearly nonexistent [19]. Nonetheless, it is well known that excessive exposure to indoor fungal bioaerosols caused by damp and mold can lead to severe health impacts, resulting in the WHO Guidelines for Indoor Air Quality on dampness and mold [133]. Climate change can alter fungal distribution and resistance patterns, and natural disasters can increase the risk of fungal outbreaks, such as mucormycosis during the 2004 Indian Ocean tsunami, due to traumatic injuries and changes in population immunity [18, 21]. Additionally, tissue damage and implantation of foreign objects due to combat-related injuries can increase the risk of fungal infections. For instance, traumatic injuries are often associated with soil-contaminated wounds, which are particularly susceptible to infections by fungi in the order Mucorales [132, 134]. Moreover, chemical residues like azole fungicide in the environment can also lead to azole persistence, resistance, or tolerance and promote fungal infections [18, 21, 134]. Therefore, it is likely (but not proven) that a coordinated approach by decision- and policy-makers, including public health sectors and One Health domains, may be helpful in mitigating the impact of social determinants. One Health solutions, in particular, can offer a comprehensive approach to tackling the rise in fungal infections, from understanding how fungicides are deposited in the natural environmental and how best to limit their usage (eg, reducing chemical residues in composted agricultural green waste) to navigating environmental/agricultural/industrial developments, practices, and pressures that drive fungicide use, implementing stricter biosecurity regulations for trade, and understanding the influence of climate change [18, 21]. Similarly, One Health programs that tackle the risk of viral infectious diseases spill over across the microorganism–environment–human interface, which means that these efforts will also influence the prevalence of viral–fungal coinfection, such as COVID-19-associated pulmonary aspergillosis or HIV-associated fungal meningitis [135, 136].

The FPPL highlights the need to implement risk-reduction strategies to reduce the emergence of AFR and IFDs, and the One Health approach provides a framework to address AFR [12]. Human health is predicated on having a stable source of nutrition, provided by agricultural systems, which necessitates the use of fungicides in the environment for the purpose of crop and horticultural protection. However, in their natural environment, opportunistic fungal pathogens are exposed to these broad-spectrum classes of antifungals, which are also used as frontline antifungal treatments in the clinic, and azole-resistant *Aspergillus fumigatus* infections have been linked to environmentally driven resistance [21]. Such dual use of near-identical antifungal chemicals in the environment and the clinic has resulted in the widespread evolution of resistance, such that azoles are increasingly failing as frontline therapy against invasive aspergillosis [21, 137]. It is likely that, owing to global variation in climates, farming practices, crops, and fungicide use, rates of environmental resistance to antifungal chemicals will vary widely; however, widescale global analyses have not yet been undertaken, and it is not clear how granular spatial data from European studies can be applied more generally [21, 137]. Concern has been voiced about the US approval of the fungicide ipflufenoquin, a dihydroorotate dehydrogenase (DHODH) inhibitor, ahead of the approval of the novel medical antifungal olorofim, which is also a DHODH inhibitor and demonstrates activity against some multidrug-resistant fungi, including azole-resistant isolates [138]. The use of fungicides such as ipflufenoquin may unintentionally cause a rise in resistant fungi in the environment, potentially rendering new antifungals ineffective [18].

Nonetheless, it is clear that in Northern Europe air exposure to azole-resistant *A. fumigatus* is widespread and that exposure to this resistant bioaerosol accounts for ∼40% of resistant infections [139, 140]. A One Health approach to mitigating the use of antimicrobials in the environment, with the aim of reducing the burden of resistance, will be critical in attempting to lessen the rising rates of resistance in human fungal pathogens [141]. Critically, a nuanced understanding of how agricultural practices may lead to selection for resistance in FPPL pathogens may also lead to methodologies to mitigate exposure [21, 137]. We recommend increasing the link between environmental risk assessments and clinical risk assessments for antifungal chemicals and aligning or upgrading policy approaches.

### Limitations of the FPPL

Fungi are an incredibly diverse group of organisms; there are thousands of known species, and many more likely exist in various ecosystems [142]. Identifying and prioritizing human pathogenic fungi are challenging tasks. Some fungal diseases that are prevalent in one region are not as prevalent in another; therefore, a single global priority list may not accurately reflect regional health priorities [12, 24]. New fungal pathogens have emerged in recent decades and are likely to continue emerging in the future; continuous surveillance will be required, and not all pathogens may be accounted for in a static list [24]. Similar to antibiotic resistance, AFR is a growing concern. Determining which resistant strains should be addressed most urgently is limited by major gaps in the data, especially in LMICs [21].

The FPPL identified major knowledge gaps in the global burden of fungal diseases [12]. Furthermore, it effectively highlights regional differences in fungal epidemiology and the need for improved surveillance, research, innovation in diagnostics, and AFR monitoring [24]. The FPPL has also assisted in promoting awareness of reference laboratory capacity in many countries and the use of AST [105, 115]. However, the FPPL has some limitations. It focuses on acute and subacute infections, with limited discussion on superficial and chronic disease. It is also limited to fungal pathogens that are associated with a serious risk of mortality or morbidity [24]. As discussed in the WHO FPPL report, some pathogens are restricted to specific geographical regions and thus might not be considered a global priority, highlighting the need for regional adaptations [12]. An example is *Paracoccidioides* spp., a pathogen that causes a high burden of disease but is confined to geographical areas such as Latin America [1, 12, 143, 144]. Similarly, we recommend adapting the list to specific patient populations; for instance, *Pneumocystis jirovecii* is ranked as a medium-priority threat on the global list, despite being one of the most common pathogens causing opportunistic infection in people with HIV/AIDS [12]. These considerations can alter the order of ranking of pathogens of concern [1, 12, 143, 144]. Any FPPL could look very different in different regions or in different patient populations. Therefore, pathogens that are ranked as medium priority or even excluded from the list should not be excluded from research or public health interventions; deprioritization could stymie R&D efforts [115].

## CONCLUSIONS

IFDs are a growing threat to global public health, and overlooking these pathogens has resulted in incorrect diagnoses, inadequate treatment, limited surveillance, elevated AFR, and major knowledge gaps in disease burden [105, 131]. The development of the FPPL remains a major step in increasing awareness of fungal pathogens, and future iterations can help to refine the list further [12, 105, 115]. The FPPL has highlighted 3 primary areas of action: strengthening laboratory capacity and surveillance, sustainable investment in R&D, and public health interventions [12]. The list can help to guide mycological laboratory capacity and training, increasing the availability of data that demonstrate risk/significance of infection [24]. Given the unmet R&D needs and perceived public health importance of fungal pathogens, the FPPL has evolved into a global initiative to systematically prioritize fungal pathogens [12, 24].

This author group believes that the FPPL can be used as a stepping stone to facilitate the prioritization of fungal pathogens from a regional perspective to guide public health interventions [12, 24]. Joint training programs can upskill clinicians and laboratory technicians in fungal disease management and improve patient outcomes, while One Health programs can reduce rates of resistance by limiting antimicrobial use. While the Northern European perspective presented here is associated with potential limitations, particularly given the distribution of fungal pathogens and approaches to tackle them can vary by geography, we believe the views highlighted in this manuscript have broader applicability beyond this region. The suggested actions and methodologies in this report can be used by policy-makers, public health experts, and other stakeholders to enhance the overall response to priority fungal pathogens, including preventing the emergence of AFR.
